# Tuberculosis in the European Union/European Economic Area: much progress, still many challenges

**DOI:** 10.2807/1560-7917.ES.2019.24.12.1900174

**Published:** 2019-03-21

**Authors:** Jean-Paul Guthmann, Walter Haas

**Affiliations:** 1 Santé publique France, Saint-Maurice, France; 2 Robert Koch Institute, Berlin, Germany

**Keywords:** tuberculosis, TB, Europe, surveillance, control

Tuberculosis (TB) is still a deadly disease in 2019. It ranks among the top 10 causes of death globally and is the most frequent cause of death from a single infectious agent. TB remains a major public health problem worldwide, with the highest disease burden in low- and middle-income countries; according to the World Health Organization (WHO), an estimated 10 million people fell ill with tuberculosis in 2017, 1.5 million of whom died [1]. Children under 5 years of age are especially at risk of developing severe disease manifestations such as meningeal or disseminated disease and are therefore at higher risk of death and sequelae. In addition, young children with untreated latent TB infection (LTBI) or disease may experience reactivated TB later in life, which poses a major obstacle for elimination of the disease.

A total of 3% of global TB cases occur in the WHO European Region [1]. From a global perspective, the European Union/European Economic Area (EU/EEA)—which comprises 31 of the 53 countries in the Region—is making much progress, with decreasing numbers of people getting sick from TB or dying from the disease [2]. Despite this improvement, several problems remain that need to be considered. In the EU/EEA, the frequency and burden of TB varies considerably between countries, with country-specific notification rates ranging from 2.6 in Liechtenstein to 66.2 per 100,000 in Romania [2]. Within the EU/EEA, TB is largely a disease of the poor, affecting the most vulnerable and impoverished population groups. In Portugal, for example, unemployed individuals were shown to be at higher risk of developing TB [3]. In France, the TB notification rate is 10 times higher in foreign-born individuals compared with the native population, and in homeless individuals this rate was 166 per 100,000 in 2015 [4]. Though the EU/EEA has a relatively low incidence of TB compared to the rest of the world, thousands of people still die from the disease every year, even though it can usually be cured by timely treatment with an adequate and complete drug regimen. The high number of multidrug-resistant (MDR) TB cases in some countries remains a constant threat for local populations and, because of travel and migration, also puts at risk those living in bordering countries and other parts of Europe. At 3.8%, the proportion of MDR TB in Germany in 2017 was almost four times higher in foreign-born individuals compared to those born in Germany (1.0%); for those born in the newly independent states of the former Soviet Union, this proportion was almost 20 times higher (19.3%) [5].

The present issue of *Eurosurveillance* features two articles that discuss issues that the EU/EEA is facing regarding TB control. The incidence of TB in most EU/EEA countries is decreasing and this is encouraging. However, one important question is whether the current rate of decrease is enough to meet the targets that all WHO Member States committed to in 2015 to reduce cases of TB and deaths from the disease [6]. This point is discussed by Merk et al., who describe the trends of TB incidence and deaths reported in the EU/EEA during the last decade [7]. The authors show that despite a clear annual decline in cases and deaths, the trend is not enough to reach the set objectives. They also point out that some countries are progressing towards ending TB faster than others, and underline the need to adapt prevention and control measures to a particular country’s situation. Despite substantial improvements in TB control in the EU/EEA, and the fact that the disease is progressively becoming rare in many countries, Merk et al. highlight that further progress in controlling and finally eliminating this severe disease will require a constant effort that is continually adapted to suit distinct geographical areas and the most affected population groups.

Treatment outcome of TB patients and factors that may influence treatment success are further key points in TB control, and these are discussed in an article by Karo et al. in this issue of *Eurosurveillance* [8]. Adequate treatment of a TB case cures the patient, rapidly limits the risk of transmission of *Mycobacterium tuberculosis* to close contacts in the family and community, and prevents the development of resistance to anti-TB drugs. Therefore, assessing patients’ treatment outcome remains essential for evaluating national TB control programs; further, identifying factors associated with an unfavourable outcome may help to target control measures in groups that are most at need. In their analysis of factors influencing treatment outcome of TB in Europe, Karo et al. showed an almost nine times higher risk of unsuccessful treatment for patients with MDR TB (Odds ratio (OR): 8.7; 95% confidence interval (CI): 5.09–14.97) [9]. The retrospective analysis in this issue used information on treatment outcome compiled by the European Centre for Disease Prevention and Control (ECDC) in the European Surveillance System (TESSy) database to investigate the association between isoniazid (INH) mono-resistance and TB treatment success. The results show that treatment success did not meet the objectives set by WHO in 2014 [6] and that treatment success for INH mono-resistant TB was significantly lower compared with fully drug-susceptible TB. The authors compare their results to those already published and conclude that increased efforts should be made towards timely detection and management of INH mono-resistant TB, which is frequently underestimated. Timely drug sensitivity testing of all cases and provision of treatment regimens adapted to the strain profile are essential components of TB control that contribute to maintaining a low prevalence of INH mono-resistance observed in the EU/EEA [10]. The timely identification and management of patients infected with a strain resistant to INH, one of the most important first-line drugs for the treatment of TB, should also prevent further development of MDR TB, which remains low overall in most countries of the EU/EEA [2].

It is clear that ending the TB epidemic poses tremendous challenges and requires a concerted international effort. Following the 1993 WHO alert declaring that TB was a global emergency [11], several initiatives (e.g. Stop TB Partnership), sources of funding (e.g. The Global Fund) and political declarations have shown that the international community is committed to the global goal of ending the TB epidemic. In November 2017, the first WHO global ministerial conference on ending TB was held in the Russian Federation and brought together 75 ministers of health, resulting in the Moscow Declaration to End TB [12]. The conference recognised that today TB is the most deadly infectious disease in the world, with considerable economic and social consequences. On 26 September 2018, the United Nations first high-level meeting on TB in New York highlighted the need for immediate action to accelerate progress towards the goal of ending the TB epidemic by 2030. In the political declaration, national leaders committed to taking specific actions against TB [13].

The elimination of TB in the EU/EEA, where a growing number of countries are progressively entering the low-incidence category, poses several challenges, as illustrated by the two articles presented in this issue of *Eurosurveillance*. The elimination of TB in this region will require additional efforts and specific actions that have been adapted to local epidemiology [14]. Treatment outcome needs to improve, particularly in drug-resistant TB cases, including cases with MDR TB, which is associated with the highest rates of unsuccessful treatment [9]; in order to achieve this, a wider use of rapid molecular testing and the adaptation of treatment regimens are necessary. Continual involvement of TB professionals and close collaboration between clinicians, microbiologists and epidemiologists working in the field are also needed. Immediate contact investigations performed around each newly detected case will help to prevent the occurrence of new cases. The use of genotyping methods to identify related cases and to understand chains of transmission should further help to get closer to a zero transmission goal.

In low-incidence countries, the majority of TB cases are generated through reactivation of latent TB infections (LTBI) acquired abroad [14]. As global migration has increased considerably in recent decades [15,16] and a significant proportion of TB cases in most EU/EEA countries are born in countries with a high incidence of TB [2], early detection and access to health services and care in this specific group should be placed as one of the top priorities of TB control. In addition, individuals with LTBI represent an important reservoir, as they may later progress to TB disease, therefore contributing to future TB burden. In the WHO European Region the prevalence of LTBI has been estimated at 13.7%; this prevalence is 0.3% for recent infections [17], which have the highest risk of progressing towards TB. An important challenge for European control programs is therefore to establish a programmatic approach to LTBI management that takes into account the TB epidemiology in various vulnerable groups, as well as the health system structure, resource allocation and political commitment [18]. This underlines that there is not one single issue to address, but rather that a strategic, comprehensive approach needs to be developed in order to meet the challenge of TB elimination.

## References

[1] World Health Organization (WHO). Global tuberculosis report 2018. Geneva: WHO; 2018. Available from: https://www.who.int/tb/publications/global_report/en/

[2] World Health Organization Regional Office for Europe (WHO/Europe)/European Centre for Disease Prevention and Control (ECDC). Tuberculosis surveillance and monitoring in Europe 2019 – 2017 data. Copenhagen: WHO/Europe; 2019. Available from: www.ecdc.europa.eu/en/publications-data/tuberculosis-surveillance-and-monitoring-europe-2019

[3] Apolinário D Ribeiro AI Krainski E Sousa P Abranches M Duarte R Tuberculosis inequalities and socio-economic deprivation in Portugal. Int J Tuberc Lung Dis. 2017;21(7):784-9. 10.5588/ijtld.16.0907 28633703

[4] Guthmann J, Aït Belghiti F, Lévy-Bruhl D. Épidémiologie de la tuberculose en France en 2015. Impact de la suspension de l’obligation vaccinale BCG sur la tuberculose de l’enfant, 2007-2015. [Epidemiology of tuberculosis in France in 2015. Impact of the suspension of the BCG vaccination obligation on childhood tuberculosis, 2007-2015]. Saint-Maurice: Santé publique France; 2017. French. Available from: http://invs.santepubliquefrance.fr/beh/2017/7/2017_7_1.html

[5] Robert Koch Institut (RKI). RKI-Bericht zur Epidemiologie der Tuberkulose in Deutschland für 2017. [RKI-report on the epidemiology of tuberculosis in Germany for 2017]. Berlin: RKI; 2018. German. Available from: https://www.rki.de/DE/Content/InfAZ/T/Tuberkulose/Archiv_Berichte_TB_in_Dtl_tab.html

[6] World Health Organization (WHO). Implementing the end TB strategy: the essentials. Geneva: WHO; 2015. Available from: http://www.who.int/tb/publications/2015/end_tb_essential.pdf?ua=1

[7] Merk H Ködmön C van der Werf MJ Will we reach the Sustainable Development Goals target for tuberculosis in the European Union/European Economic Area by 2030? Euro Surveill. 2019;24(12):1900153 10.2807/1560-7917.ES.2019.24.12.1900153 PMC6440579 30914077

[8] Karo B Kohlenberg A Hollo V Duarte R Fiebig L Jackson S Isoniazid (INH) mono-resistance and tuberculosis (TB) treatment success: analysis of European surveillance data, 2002 to 2014. Euro Surveill. 2019;24(12):1800392 10.2807/1560-7917.ES.2019.24.12.1800392 PMC6440580 30914081

[9] Karo B Hauer B Hollo V van der Werf MJ Fiebig L Haas W Tuberculosis treatment outcome in the European Union and European Economic Area: an analysis of surveillance data from 2002-2011. Euro Surveill. 2015;20(49):30087. 10.2807/1560-7917.ES.2015.20.49.30087 26676247

[10] van der Werf MJ Ködmön C Hollo V Sandgren A Zucs P Drug resistance among tuberculosis cases in the European Union and European Economic Area, 2007 to 2012. Euro Surveill. 2014;19(10):20733. 10.2807/1560-7917.ES2014.19.10.20733 24650865

[11] World Health Organization (WHO). TB: a global emergency. WHO report on the TB epidemic. Geneva: WHO; 1994. Available from: http://apps.who.int/iris/handle/10665/58749

[12] World Health Organization (WHO). New Global Commitment to fight tuberculosis. Geneva: WHO; 2017. Available from: https://www.who.int/news-room/detail/17-11-2017-new-global-commitment-to-end-tuberculosis

[13] World Health Organization (WHO). About the UN high-level meeting. Geneva: WHO; 2018. Available from: https://www.who.int/news-room/events/un-general-assembly-high-level-meeting-on-ending-tb/about-the-un-high-level-meeting

[14] Lönnroth K Migliori GB Abubakar I D’Ambrosio L de Vries G Diel R Towards tuberculosis elimination: an action framework for low-incidence countries. Eur Respir J. 2015;45(4):928-52. 10.1183/09031936.00214014 25792630 PMC4391660

[15] Hargreaves S Lönnroth K Nellums LB Olaru ID Nathavitharana RR Norredam M Multidrug-resistant tuberculosis and migration to Europe. Clin Microbiol Infect. 2017;23(3):141-6. 10.1016/j.cmi.2016.09.009 27665703

[16] Annesi-Maesano I Ringing the alarm bells about migrants’ health. Int J Tuberc Lung Dis. 2018;22(2):123-4. 10.5588/ijtld.17.0872 29506607

[17] Houben RM Dodd PJ The Global Burden of Latent Tuberculosis Infection: A Re-estimation Using Mathematical Modelling. PLoS Med. 2016;13(10):e1002152. 10.1371/journal.pmed.1002152 27780211 PMC5079585

[18] Rosales-Klintz S Bruchfeld J Haas W Heldal E Houben RMGJ van Kessel F Guidance for programmatic management of latent tuberculosis infection in the European Union/European Economic Area. Eur Respir J. 2019;53(1):1802077. 10.1183/13993003.02077-2018 30655449

